# Distinct risk factors for obsessive and compulsive symptoms in chronic schizophrenia

**DOI:** 10.1017/S003329171800017X

**Published:** 2018-02-19

**Authors:** Emilio Fernandez-Egea, Yulia Worbe, Miguel Bernardo, Trevor W. Robbins

**Affiliations:** 1 Clozapine Clinic, Cambridgeshire and Peterborough NHS Foundation Trust, Cambridge, UK; 2 Department of Psychiatry, University of Cambridge, Cambridge, UK; 3 Behavioural and Clinical Neuroscience Institute, University of Cambridge, Cambridge, UK; 4 Centro de Investigación Biomédica en Red de Salud Mental (CIBERSAM), Barcelona, Spain; 5 Sorbonne Université, 75005 Paris, France; Department of Physiology, Neurophysiology Unit, Hospital Saint-Antoine, Paris, France; Institute du Cerveau et de Moelle Epigniere, Paris, France; 6 Barcelona Clinic Schizophrenia Unit, Neuroscience Institute, Hospital Clinic of Barcelona, Barcelona, Spain; 7 Institut d'Investigacions Biomèdiques August Pi i Sunyer (IDIBAPS), Barcelona, Spain; 8 Department of Psychiatry, University of Barcelona, Barcelona, Spain; 9 Department of Psychology, University of Cambridge, Cambridge, UK

**Keywords:** Antipsychotics, habit formation, clozapine, schizophrenia, obsessive compulsive disorder.

## Abstract

**Background:**

Obsessive-compulsive disorder (OCD) is common in clozapine-treated patients although the actual prevalence, phenomenology and risk factors remain unclear. The aim of the present study was to address the three aforementioned questions.

**Methods:**

The electronic records of a large cohort of clozapine-medicated schizophrenia patients routinely screened for OCD were used. The Obsessive Compulsive Inventory Revised version (OCI-R) was available from 118 cases and a 21 points cut-off threshold for OCD was defined.

**Results:**

OCD prevalence was 47%, higher in patients on poly-pharmacy than on monotherapy (64% vs 31%; p = 0.001). Two OCI-R factors had significantly higher scores and distinct risk factors: checking behaviour (mean = 5.1; SD = 3.6) correlated with length of clozapine treatment (r = 0.21; p = 0.026), and obsessing factor (mean = 4.8; SD = 3.6) correlated with psychosis severity (r = 0.59; p = 0.001). These factors along with total OCI-R, did not correlate with either clozapine dose or plasma levels, after correcting for psychosis severity.

**Conclusions:**

Screening for OCD in clozapine patients, and probably in those treated with structurally similar drugs with potent antiserotoninergic properties, should be widely adopted by clinicians. Further research is needed to understand the pathophysiology underlying repetitive behavior onset in clozapine-treated patients.

## Background

Obsessive–compulsive disorder (OCD) is common in schizophrenia patients treated with antipsychotics with significant anti-serotoninergic action (Swets *et al.* 2014; Grillault Laroche & Gaillard, 2016). Clozapine was the first drug reported (Baker *et al.* 1992) and is still the medication more frequently associated with OCD (Poyurovsky *et al.* 2001), along with olanzapine (Schirmbeck *et al.* 2011). However, the prevalence, phenomenology and pathophysiology of clozapine-induced OCD remain unclear.

In the literature, the prevalence of clozapine-induced OCD ranges from <5% (Mahendran *et al.* 2007) to over 70% (Schirmbeck *et al.* 2011). This discrepancy reflects the heterogeneity in the methodology used, such as the diagnostic instrument (structured clinical interview *v.* scale score threshold) and the sample characteristics, among others. For instance, some studies only included patients on clozapine monotherapy (Lin *et al.* 2006; Schirmbeck *et al.* 2011), whereas others broaden the scope to cases using concomitant medication (Poyurovsky *et al.* 2001; Mukhopadhaya *et al.* 2009). Noteworthy, medications like antidepressants or antipsychotics are also prescribed for treating OCD and therefore could potentially mask OCD prevalence and severity.

From the point of view of phenomenology, degree of insight should help to distinguish between delusions and obsessions (Poyurovsky *et al.* 2004), but this can be difficult to disentangle in the presence of chronic positive symptoms in clinical practice (Leung & Palmer, 2016). Most studies of prevalence have not taken into account psychosis severity (Poyurovsky *et al.* 2001; Lin *et al.* 2006; Faragian *et al.* 2009; Mukhopadhaya *et al.* 2009). When psychosis is considered, most (Guillem *et al.* 2009; Sa *et al.* 2009) but not all (Faragian *et al.* 2009) studies have associated delusions and OCD severity, which suggests an overlap between them. Clinically, a predominantly compulsion profile was initially described in clozapine-associated OCD (Ongür & Goff, 2005; Guillem *et al.* 2009; Kim *et al.* 2012), but this has not always been replicated (Schirmbeck *et al.* 2011; Doyle *et al.* 2014). The type of compulsion also varies across studies, as the predominance of checking (Poyurovsky *et al.* 2004; Mukhopadhaya *et al.* 2009; Schirmbeck *et al.* 2011) and also cleaning and washing (Poyurovsky *et al.* 2001; Faragian *et al.* 2009; Guillem *et al.* 2009) compulsions have been reported. Nevertheless, there is a high concordance of the obsession–compulsion pair: contamination with washing (Poyurovsky *et al.* 2001; Ongür & Goff, 2005; Faragian *et al.* 2009; Sa *et al.* 2009) and doubting with checking (Leung & Palmer, 2016).

Length of clozapine treatment is a replicated risk factor for developing OCD (Lin *et al.* 2006; Boyette *et al.* 2011; Schirmbeck *et al.* 2011; Scheltema Beduin *et al.* 2012). However, the evidence for OCD being a clozapine dose-dependent side effect is still inconclusive. For instance, one study found that patients with clozapine-associated OCD were treated with higher drug doses (Mukhopadhaya *et al.* 2009), and another study (Schirmbeck *et al.* 2011) showed a correlation between dose and OCD symptoms but failed to correlate with clozapine plasma levels. Noteworthy, plasma level is considered as a more accurate measure, as it is not affected by patients’ compliance or pharmacokinetic factors (i.e. smoking). To date, no study has found a correlation between clozapine plasma levels and obsessive and compulsive symptom (OCS) severity (Lin *et al.* 2006; Schirmbeck *et al.* 2011).

### Aims of the study

The aims of this study was to address three aforementioned questions of OCD prevalence, clinical profile and associated severity factors, using electronic records of a large cohort of clozapine-medicated schizophrenic patients. Patients were routinely screened for OCD using standardised scales as well as relevant clinical, psychometric and demographic data.

## Material and methods

### Study design

This is a cross-sectional, single-centre study of a cohort of clozapine-treated schizophrenia patients at the Cambridgeshire and Peterborough NHS Foundation Trust, a community mental health trust in the UK. The study included electronic clinical records of more than 200 cases from August 2015 to June 2017. The anonymised clinical records were embedded in an ethically approved database for research and clinical purposes (13/EE/0121). All cases were reviewed by the same care consultant (EFE).

### Study sample

All records of clozapine-treated schizophrenia cases were initially included, regardless of other medication status. The only exclusion criterion was not suffering with a primary psychotic disorder (i.e. off label use of clozapine). All cases were included for the prevalence and phenomenology analyses. Stricter criteria were defined for the analysis of the role of clozapine dose and plasma level on OCD severity, i.e: only cases on clozapine monotherapy, with clozapine plasma levels available and with evidence of adequate medication compliance measured by the clozapine:norclozapine ratio, were included.

### Clinical assessment

Assessment includes full psychiatric history, mental state examination, current medication list, smoking, legal and illegal drugs habits, comprehensive clozapine side effects assessment, physical health assessment and psychopathological scales (see below), among others. Per protocol, clozapine and norclozapine plasma levels are assessed every 3 years, unless otherwise required for clinical reasons (i.e. change in smoking status, side effects or compliance queries). Levels were determined at the Toxicology Department, King's College Hospital, London, UK.

### Psychopathological scales

All assessment included Global Assessment of Functioning (GAF) and Clinical Global Impression for Schizophrenia (Haro *et al.* 2003), which measures severity of positive, negative, depressive, cognitive symptoms domains as well as an overall score, all ranging from 1 (no symptoms) to 7 (extremely severe). The Short version of the Warwick-Edinburg Wellbeing scale was performed annually (Brown *et al.* 2016).

Since August 2015, the Obsessive Compulsory Inventory-Revised (OCI-R) (Foa *et al.* 2002) was added to the routine assessment while subjects were doing the routine plasma level control, in order to screen subjects with obsessive symptoms that might require intervention. OCI-R is a widely used 18-item self-rated questionnaire easy to implement in clinical settings. Each question has a five-point Likert-type score that measures the degree of distress or being bothered with common OCD phenomena, ranging from 0 (‘not at all’) to 4 (‘extremely’). The cut-off point for OCD diagnosis is 21 for the total OCI-R. The scale is composed of six factors of three items each, evaluating washing behaviour, obsessing thinking, hoarding, ordering, checking behaviour and mental neutralisation. For OCD diagnosis, a cut-off point for the obsessive factor of 5 has been defined (Foa *et al.* 2002). As there are no specific cut-off points for the other five factors, here we have used the same five-point cut-off for clinically significant symptoms to reach the disorder threshold.

### Concomitant medication assessment

Poly-pharmacy is common in treatment-resistant schizophrenia. Some of these drugs can potentially influence obsessive symptoms. For instance, clozapine augmentation with aripiprazole is common for persistent psychotic symptoms (Cardinal *et al.* 2015) but can also be added as therapy for OCD (Englisch *et al.* 2009; Eryılmaz *et al.* 2013), sedation (Perdigués *et al.* 2016) or metabolic complications (Zimbron *et al.* 2016). Similarly, antidepressants are primarily used for depression, anxiety or OCD. Rather than excluding cases on poly-pharmacy, we adopted a pragmatic approach by recording dichotomic (yes/no) variable for medication use, regardless of the primary indication: use of any drug with potential effect on OCD (aripiprazole, selective serotoninergic reuptake inhibitor, SSNRI or tricyclics), which for simplification in this manuscript we termed as anti-OCD medication.

### Statistical analysis

All statistical analyses were conducted using SPSS v23.0 for Mac, with a two-tail 0.05 significance level. As described in the Results section, χ^2^, Student's *t* test, analysis of variance and bivariate correlation using Pearson *r* coefficient were used according to the analysis needs. Partial correlation was used for controlling the role of a third factor on bivariate correlations. An exploratory factor analysis was also carried out to compare our data structure with the six-factor component of the OCI-R.

## Results

Two hundred and one patients on clozapine and with primary diagnosis of non-affective psychosis were included. Of those, 122 (60.7%) completed the OCI-R scale during the clinical review. Three patients were excluded from the analyses as OCD predated psychosis (two cases) and another case due to lack of reliability in the answers, reaching a final sample of 118 cases.

### Prevalence of OCD

Among the 118 cases, the mean OCI-R total score was 20.3 (s.d. = 13.7) and 47% (*n* = 55) reached threshold criteria for OCD. Fifty-five of the 118 cases (47%) were on anti-OCD medication. As shown in Table 1, patients on poly-pharmacy had significantly higher OCI-R scores (24.6 s.d. = 13.2 *v.* 16.2 s.d. = 12.2; *t* = 3.465; *p* = 0.001) and OCD prevalence (64% *v.* 31%; χ^2^ = 12.001; *p* = 0.001) as well as higher clinical global impression (CGI)-positive scores (3.1 s.d. = 1.6 *v.* 2.5 s.d. = 1.5; *t* = 2.184; *p* = 0.031).

**Table 1.** Samples sociodemographic and clinical variables, expressed as mean and standard deviation

	Total sample	Monotherapy	Poly-therapy	*p*
N	118	63	55	
Gender male n (%)	98 (83%)	54 (85.7%)	44 (80.0%)	0.28
Age (in years)	44.3 (11.0)	43.3 (12.1)	45.5 (9.7)	0.29
Clozapine treatment (in years)	14.6 (7.1)	14.6 (7.4)	14.5 (6.8)	0.93
Clozapine dose (mg/day)	346 (141)	337.1 (144.7)	356.8 (138.3)	0.45
Clozapine plasma level (mg/dL)	0.42 (0.22)	0.39 (0.22)	0.45 (0.23)	0.20
Norclozapine plasma levels (mg/dL)	0.27 (0.13)	0.26 (0.13)	0.29 (0.13)	0.28
GAF score	70.1 (12.9)	71.5 (14.0)	68.4 (11.4)	0.19
CGI-positive	2.8 (1.6)	2.51 (1.5)	3.13 (1.6)	**0.03**
CGI-negative	3.0 (1.3)	2.98 (1.4)	3.09 (1.2)	0.65
CGI-overall	2.7 (1.0)	3.27 (1.3)	3.67 (1.0)	0.22
On aripiprazole	24%		51%	
On antidepressants (SSRI/SNRI)	33%		71%	

*p* represents *p*-significance group differences (monotherapy *v.* poly-therapy) using Student's *t* or χ^2^ tests.

SSRI, selective serotoninergic reuptake inhibitor (fluoxetine, paroxetine, fluvoxamine, citalopram, sertraline and escitalopram); SNRI, serotonin and norepinephrine reuptake inhibitors (venlafaxine and duloxetine).

### OCS profile

We tested if our OCI-R responses fit the original OCI-R factor structure, as its validity has not been formally tested before in schizophrenia patients. To this aim, we used an exploratory factor analysis of the individual answers, fixing a six-factor model and choosing a maximum likelihood as extraction method with no rotation. A χ^2^ measure of goodness-of-fit, testing the null hypothesis that six factors were adequate to explain 76% of the co-variances in our data, showed a χ^2^ = 112.67, with a *p* < 0.001.

**Fig. 1.**
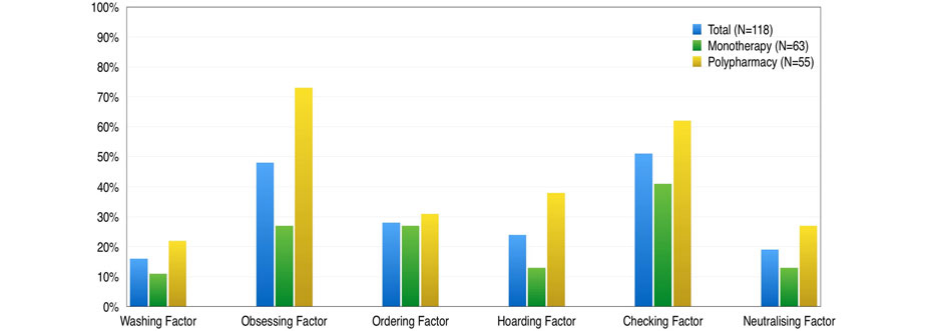
Percentage of significant (cut-off point of 5) of the six OCI-R factors, in the total sample (dark grey), monotherapy (light grey) and poly-pharmacy (black) groups.

Mean scores of the six factors were (from higher to lower): checking 5.1 (s.d. = 3.6); obsessing 4.8 (s.d. = 3.6); hoarding 3.2 (s.d. = 3.2); ordering 2.9 (s.d. = 3.0); neutralising 2.4 (s.d. = 3.0) and washing 1.8 (s.d. = 2.4). All six factors showed significant greater severity in the poly-medicated group (Table 2). When a cut-off point of 5 was applied to indicate ‘disorder’, about half of patients reported significantly distressing checking compulsions and obsessive thinking (Fig. 1). In order to improve readability, we will report here only checking and obsessing factors, although online Supplementary Table shows analyses with all six factors.

**Table 2.** Mean score and standard deviation (s.d.) of the OCI-R total and six factors

	Total sample	Monotherapy	Polypharmacy	*p* significance
Sample size	118	63	55	
OCI-R total score	20.3 (13.7)	16.5 (12.2)	24.6 (13.2)	**0**.**001**
Checking factor	5.1 (3.6)	4.3 (3.4)	5.8 (3.6)	**0.018**
Obsessing factor	4.8 (3.6)	3.4 (3.1)	6.5 (3.5)	**<0.001**
Hoarding factor	3.2 (2.8)	2.4 (2.5)	4.1 (2.9)	**0.001**
Ordering factor	2.9 (3.0)	2.8 (2.9)	3.1 (3.1)	0.614
Neutralising factor	2.4 (3.0)	2.0 (2.6)	2.8 (3.3)	0.133
Washing factor	1.8 (2.4)	1.5 (2.1)	2.2 (2.8)	0.137

*p* Significance indicates the Stuedent's *t* test comparison between clozapine monotherapy and poly-pharmacy groups.

### Role of severity of schizophrenia-positive symptoms in OCS

OCI-R is a self-rated questionnaire and some questions illustrate the overlap of obsessive and psychotic phenomena. For instance, question 6 (obsessing factor) states: ‘I find difficult to control my own thoughts’. In turn, some positive symptoms of schizophrenia might lead to patient's repetitive behaviour (Faragian *et al.* 2009), so we explored the effect of psychosis on the OCI-R sub-scales.

In the monotherapy sub-sample, psychosis severity (measured with CGI-positive symptoms score) significantly correlated with total OCI-R (*r* = 0.38; *p* = 0.002) and obsessing factor (*r* = 0.59; *p* < 0.001) but not with checking behaviour factor (*r* = 0.17; *p* = 0.18). Similar results were seen in the poly-medicated group, where psychosis significantly correlated with total OCI-R (*r* = 0.32; *p* = 0.018) and obsessing factor (*r* = 0.46; *p* < 0.001) but not checking factor (*r* = 0.20; *p* = 0.15).

We further explored the role of psychosis by dividing the 118 cases into those with positive symptoms (GCI-positive symptoms >1; *n* = 84) and those with complete psychosis remission (GCI-positive symptoms = 1; *n* = 34). Patients with active psychosis presented a significantly higher total OCI-R score (22.5 s.d. = 13.5 *v.* 14.8 s.d. = 10.9; *t* = 2.955; *p* = 0.004) and higher obsessing factor (5.9 s.d.= 3.5 *v.* 2.3 s.d. = 2.5; *t* = 5.277; *p* < 0.001) but not checking factor (5.4 s.d. = 3.7 *v.* 4.3 s.d. = 3.2; *t* = 1.542; *p* = 0.126). The result made us consider positive symptoms severity as a confounding factor that should be taken into consideration in subsequent analyses.

### Factors associated with OCD

We assessed length of clozapine treatment and degree of clozapine exposure as risk factors for OCD onset, as both have been described in the literature (Lin *et al.* 2006; Boyette *et al.* 2011; Schirmbeck *et al.* 2011; Scheltema Beduin *et al.* 2012).

**Fig. 2.**
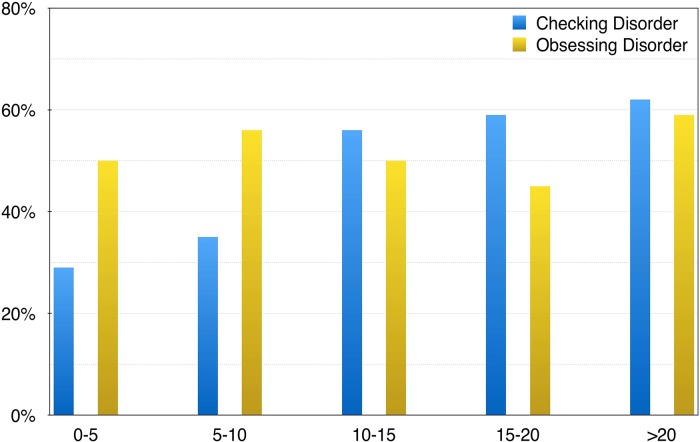
Percentage of significant ‘obsessive’ and ‘checking behaviour’ using the OCI-R factor and a five-point cut-off threshold and length of clozapine treatment in 5-year intervals.

In our sample, length of clozapine treatment significantly correlated with checking severity (*r* = 0.21; *p* = 0.026), but not with total OCI-R (*r* = 0.15; *p* = 0.10) or obsessing factor (*r* = 0.02; *p* = 0.86). A partial correlation of length of treatment and checking factor, controlled by psychosis severity, showed a stronger correlation (*r* = 0.25; *p* = 0.006). Figure 2 plots length of clozapine treatment and significant checking and obsessing factors (cut-off point ⩾5).

With regard to the influence of clozapine dose on OCD, we controlled for three confounders that have not been considered together before: (i) clozapine plasma levels, as dose is markedly influenced by pharmacokinetic factors (i.e. smoking) (Couchman *et al.* 2010); (ii) poor compliance with medication, which can be irregular so we used the plasma clozapine:norclozapine ratio, as it is a proxy for compliance (Couchman *et al.* 2010) and (iii) as in our sample, positive symptoms significantly correlated with clozapine dose (*r* = 0.22; *p* = 0.015) and plasma levels (*r* = 0.25; *p* = 0.007), we used partial correlation to co-variate for psychosis severity.

Therefore, for this analysis, we included cases on monotherapy, with no change in dose or smoking habit during the prior 3 months and showing adequate medication compliance, measured as clozapine:norclozapine ratio between 0.5 and 1.0, to a final sample of 37. Table 3 shows the direct correlation between OCS and clozapine dose and plasma levels as well as partial correlation of these parameters controlling for psychosis.

**Table 3.** Correlation of clozapine dose, plasma levels and norclozapine *v*. total OCI-R score and obsessing and checking factor in 37 cases on clozapine monotherapy and adequate compliance

	Direct correlation			Correlation mediated by positive symptoms		
	Clozapine dose	Clozapine plasma level	Norclozapine plasma levels	Clozapine dose	Clozapine plasma level	Norclozapine plasma levels
OCI-R total score	*R* = 0.14; *p* = 0.40	*R* = 0.29; *p* = 0.08	*R* = 0.25; *p* = 0.14	*R* = −0.02; *p* = 0.89	*R* = −0.06; *p* = 0.71	*R* = −0.11; *p* = 0.54
Obsessing factor	*R* = 0.18; *p* = 0.28	***R* = 0.46;** ***p* = 0.004**	***R* = 0.37;** ***p* = 0.024**	*R* = 0.09; *p* = 0.60	*R* = 0.22; *p* = 0.19	*R* = 0.25; *p* = 0.14
Checking factor	*R* = −0.01; *p* = 0.96	***R* = 0.33;** ***p* = 0.048**	*R* = 0.29; *p* = 0.08	*R* = 0.10; *p* = 0.57	*R* = 0.18; *p* = 28	*R* = 22; *p* = 0.19

First using bivariate correlation and then a partial correlation mediated by psychosis severity.

## Discussion

In our study, with the largest sample studied so far, about half of the 118 cases of clozapine-treated schizophrenia patients reported OCD measured with the OCI-R. Crucially, we were able to identify the distinct risk factors associated with the two main OCD subscales. Checking compulsions, the most prevalent group of symptoms, were associated with the length of clozapine treatment, whereas obsessions, the second most common group of symptoms, were associated with psychosis severity. None of the symptoms were associated with clozapine dose or plasma level.

The British National Formulary still considers OCD as a rare (one out of 10 000) clozapine side effect. In our sample, the OCS prevalence was 47%, a mid-point between 5% and 74% (de Haan *et al.* 1999; Lin *et al.* 2006; Mahendran *et al.* 2007; Mukhopadhaya *et al.* 2009; Schirmbeck *et al.* 2011) among the reported ranges in clozapine-treated patients. This prevalence is three times the OCD prevalence in non-clozapine patients (Swets *et al.* 2014) and suggests a stronger effect in clozapine-treated patients. To limit a potential prevalence overestimation, we used a 21-point cut-off for OCD diagnosis, instead of the less restrictive 18 points suggested by OCI-R (Foa *et al.* 2002). Consistent with previous reports (Guillem *et al.* 2009), we also found a higher prevalence in the poly-medicated and more severe cases (64%) compared with those with monotherapy (31%). In summary, our results might help to raise awareness of OCD in clozapine-treated patients among clinicians.

Importantly, independent OC factors were adequately measured with the OCI-R, as seen in the contrast of the six-factor structure in our sample and the original OCI-R validation (Foa *et al.* 2002), which is relevant taking into account it is a self-rated scale. This is in line with prior factor analyses using Yale-Brown Obsessive-Compulsive Scale (Y-BOCS) (Goodman *et al.* 1989) where the same five-factor structure was found in 51 cases of antipsychotic-induced OCD (Kim *et al.* 2012) and in 110 cases of comorbid OCD in schizophrenia (Faragian *et al.* 2009). Our findings support the use of standardised OCD symptom scales in clinical settings for screening and monitoring of OCS in this population (Doyle *et al.* 2014; Swets *et al.* 2014).

Clinically, we found a shift towards predominantly compulsive behaviours after prolonged (a decade) treatment with clozapine. Checking was the most prevalent OCD symptom with half of patients reporting significant distress, which is consistent with prior studies in both clozapine- (Kim *et al.* 2012) and non-clozapine-treated (Faragian *et al.* 2009) patients. In our sample, checking gradually increased over time (Fig. 2), with 29% prevalence among the cases with <5 years of treatment to 69% in those with more than 20 years of treatment. This replicates prior reports (Lin et al. 2006; Boyette *et al.* 2011; Schirmbeck *et al.* 2011; Scheltema Beduin *et al.* 2012) associating longer clozapine exposure to more checking compulsion.

We also found that obsessing was associated with severity of positive symptoms, but not with treatment duration or clozapine dose/plasma level. Our results replicate prior studies using Y-BOCS that shown a specific correlation between delusion severity and obsessions (Guillem *et al.* 2009; Sa *et al.* 2009). A study also using OCI-R (Kumbhani *et al.* 2010) did not report a correlation, but included only 29 patients on full remission of psychosis, which can account for the difference in the findings.

Nevertheless, discerning whether an unwanted thought is an obsession or a delusion with partial insight in a patient with active psychosis can be on occasion clinically challenging but also relevant for treatment decisions (Leung & Palmer, 2016). Of note, these conceptual debates are not exclusive to schizophrenia. For instance, the distinction of whether specific repetitive behaviour represents complex tics or compulsions in Tourette disorder have been studied (Kim *et al.* 2012) and about 20% of cases present both symptoms at the same time. For our study, we decided a pragmatic approach, measuring both phenomena independently, whereas others have excluded subjects, in whom OCD symptoms might be attributed to psychotic experience (Poyurovsky *et al.* 2001; Poyurovsky *et al.* 2004). The OCI-R factor structure in our sample mimics the six factors structure in pure OCD cases, and therefore we are relatively confident that the obsessing factor was adequately measured. However, further study of the overlapping phenomenology is required.

To date, no studies had found a direct correlation between clozapine plasma levels and OCD symptoms or severity. In one study of 26 patients on clozapine monotherapy (Schirmbeck *et al.* 2011), a correlation of OCD severity with dose, but not plasma levels was found. Significantly, dose is not an accurate measure of exposure to clozapine, as this is also influenced by compliance or pharmacokinetic factors. Another study showed that clozapine schizophrenia patients with OCD had higher clozapine plasma levels (Lin *et al.* 2006). However, this study did not account for other pharmacological interactions as clozapine patients with OCD group were treated with fluvoxamine, an antidepressant for OCD that significantly increases clozapine plasma levels (Couchman *et al.* 2010). Finally, none of these studies controlled for psychosis severity, which in our sample strongly correlated with clozapine dose. We initially found a direct bivariate correlation between plasma levels and obsessing and checking behaviour. However, this correlation became non-significant when controlled by level of psychosis (see Table 3), suggesting that severity of positive symptom could be a confounding factor.

Nevertheless, the absence of correlation of clozapine dose and OCD in our study should be considered cautiously as these results relied on a cross-sectional evaluation. Indeed, some previous findings also pointed to OCD improvement after clozapine reduction or withdrawal (Englisch *et al.* 2009; Leung & Palmer, 2016). A potential explanation would be that OCD symptoms were triggered by a high clozapine dose. If the dose was later reduced due to other factors (i.e. sedation or metabolic side effects), we would not have seen a correlation in our study. A long-term follow-up study before and after clozapine would help to understand this particular aspect.

The pathophysiological mechanisms of clozapine-induced OCD are beginning to be elucidated. Recent studies support a biological plausibility based on similarities in cognitive deficits (Patel *et al.* 2010; Schirmbeck *et al.* 2011), brain circuits (Schirmbeck *et al.* 2015) and genetic variants (Kwon *et al.* 2009) to those with pure OCD. Specifically, similarly to pure OCD patients, clozapine-treated schizophrenia patients with OCD consistently showed abnormal impulse inhibition, a tendency to response perseveration and difficulties in set-shifting abilities (Patel *et al.* 2010; Schirmbeck *et al.* 2011) associated with abnormal activation of orbitofrontal cortex (Schirmbeck *et al.* 2015) and linkage with significant variants of the SLC1A1 gene (Kwon *et al.* 2009).

The nature of the compulsive symptoms arising from chronic clozapine treatment, with its predominance in checking compulsion and obsessions, is a relevant observation. We would like to hypothesise a cognitive interpretation. In our sample, the two symptoms were associated with distinct risk factors: ‘obsessing’ correlated with positive symptoms and ‘checking’ with longer treatment. We suggest that abnormal habitual *v.* goal-directed behavioural control might be a coherent explanation as it integrates the content of psychotic symptoms and clozapine's mechanism of action. Briefly, the abnormal habit-formation model posits that OCD might result from an imbalance between excessive formation of stimulus–response associations and a diminished ability for goal-directed actions (Gillan *et al.* 2016). Thus, repetition in the context of a diminished ability for consideration of action outcome, leads to automatisation of behaviour. Indeed, schizophrenia patients exhibit deficits in goal-directed actions, likely representing a failure of cortical control over striatum (Morris *et al.* 2015), which might also increase vulnerability to OCD.

Using this conceptual framework, our hypothetical model suggests two OCD stages in clozapine-treated schizophrenia patients. Initially, goal-directed checking behaviour would be driven by psychosis (i.e. checking due to delusional hypervigilance). Then, a second phase characterised by ‘obsessions’ ameliorated by clozapine treatment but with a persisting (habitual) repetitive checking behaviour. A plausible mechanism for the persistence of repetitive behaviour would be clozapine's antagonism of 5-HT_2A_ and 5-HT_2C_ receptors (Schirmbeck & Zink, 2012). Decrease in serotonin neurotransmission in the brain causes perseveration in reversal learning tasks, the hallmark of cognitive inflexibility in compulsive behaviours including OCD (Robbins *et al.* 2012). For instance, in non-human primates, selective reduction in prefrontal serotonin is associated with cognitive inflexibility (Clarke *et al.* 2004). Similarly in rodents either reduction of 5-HT_2A_ binding in the orbitofrontal cortex (Barlow *et al*. 2015) or 5-HT_2A_ receptor antagonism (Boulougouris *et al*. 2008) is associated with impaired reversal learning. In humans, a dietary tryptophan depletion (a technique that acutely reduces serotonin in the brain) promotes habitual over goal-directed control (Worbe *et al.* 2015) and affects goal-directed learning (Worbe *et al.* 2016). Moreover, schizophrenia patients on clozapine who develop OCD present selective perseveration, as seen in the extra-dimensional set-shifting task (Patel *et al.* 2010). Taken together, these results suggest several plausible mechanisms such as impaired cognitive flexibility, impaired goal-directed behavioural control and reliance on habits by which clozapine antagonism to serotonin receptors might induce repetitive behaviour. This, our model integrates not only the present results but also the results of previous studies (Lin *et al.* 2006; Boyette *et al.* 2011; Schirmbeck *et al.* 2011; Scheltema Beduin *et al.* 2012; Schirmbeck & Zink, 2012) and patients’ narratives. Others have described that non-clozapine patients present a profile characterised by higher contamination obsessions and washing compulsions (Krüger *et al.* 2000; Faragian *et al.* 2009), consistent with the proposed model of abnormal habit formation.

There are limitations to our study. The first limitation is the inability to replicate our findings experimentally on healthy volunteers. As a drug with potentially lethal side effects, clozapine cannot be tested in healthy volunteers and it is only available for severe cases of schizophrenia, which limits experimental studies in humans. Second, despite that our sample is the largest studied to date, due to the complexity of the disorder, the amount of confounding factors and the staged approach proposed, a replication using an even larger sample is warranted. Additionally, this was a cross-sectional study, whereas a prospective design would address some of limitations of our approach such as the specific time of onset of checking behaviour. However, the feasibility of such study might be compromised by the fact that we found that OCD prevalence increased only after 10 years treatment with the same medication. Finally, OCD symptoms were evaluated using a self-rated scale and the study might benefit of replication using a clinician-rated scale such as Y-BOCS. However, we found that the responses followed the five-factor structure, suggesting that the answers were, at least, similar to other population such as OCD patients. There are some other inherent limitations related to the current diagnostic classification, as perhaps using two distinct nosologic categories (schizophrenia and OCD) does not fully capture or explain the phenomena. In line with the recent National Institute of Mental Health (NIHM) Research Domain Criteria (RDoC) initiative (Insel, 2014), checking behaviour in clozapine-treated patients might be better characterised in schizophrenia patients as a drug-enhanced habit, possibly mediated by the striatum.

## Supplementary material

For supplementary material accompanying this paper visit http://dx.doi.org/10.1017/S003329171800017X. click here to view supplementary material
