# Supplementary material for: Distinct risk factors for obsessive and compulsive symptoms in chronic schizophrenia
Source: Psychol Med. 2018 Feb 19;48(16):2668–75. doi: 10.1017/S003329171800017X (PMC6236440; doi:10.1017/S003329171800017X)
Supplement: Supplementary file 1 [file S003329171800017Xsup001.docx]

**Table A (Supplementary)**. Correlation of main variables (clozapine dose and plasma levels, psychosis severity and time on clozapine) with total OCI=R scale and its six factors.

|  | Clozapine dose | Clozapine plasma level | Norclozapine plasma levels | Psychosis Severity | | Time on clozapine | |
| --- | --- | --- | --- | --- | --- | --- | --- |
|  |  |  |  | Monotherapy | Poly-therapy | Monotherapy | Poly-therapy |
| Sample size | 37 | 37 | 37 | 63 | 55 | 63 | 55 |
| OCI-R total score | R=.14;  p=.40 | R=.29;  p=.08 | R=.25;  p=.14 | **R=.38;**  **p=.002** | **R=.32;**  **p=.018** | R=.20;  p=.11 | R=.11;  p=.41 |
| Washing Factor | R=.09;  p=.59 | R=-.09;  p=.60 | R=-.17;  p=.32 | R=.20;  p=.11 | R=.01;  p=.48 | R=-.04;  p=.74 | R=-.15;  p=.26 |
| Obsessing Factor | R=.18;  p=.28 | **R=.46;**  **p=.004** | **R=.37;**  **p=.024** | **R=.59;**  **P<.001** | **R=.46;**  **P<.001** | R=-.01;  p=.99 | R=.05;  p=.73 |
| Hoarding Factor | R=.09;  p=.60 | R=.23;  p=.16 | R=.15;  p=.38 | **R=.33;**  **p=.008** | R=.13;  p=.35 | **R=.25;**  **p=.050** | R=-.01;  p=.95 |
| Ordering Factor | R=.27;  p=.10 | R=.20;  p=.23 | R=.17;  p=.31 | R=.18;  p=.15 | R=.18;  p=.19 | R=.07;  p=.56 | R=.03;  p=.816 |
| Checking Factor | R=-.01; p=.96 | **R=.33;**  **p=.048** | R=.29;  p=.08 | R=.17;  p=.18 | R=.20;  p=.15 | **R=.360;**  **p=.004** | R=.04;  p=.76 |
| Neutralizing Factor | R=.21;  p=.20 | R=.06;  p=.70 | R=.16;  p=.31 | R=.19;  p=.12 | R=.21;  p=.13 | R=.20;  p=.11 | R=.20;  p=.14 |
